# Climatic and topographic variables control soil nitrogen, phosphorus, and nitrogen: Phosphorus ratios in a *Picea schrenkiana* forest of the Tianshan Mountains

**DOI:** 10.1371/journal.pone.0204130

**Published:** 2018-11-01

**Authors:** Zhonglin Xu, Yapeng Chang, Lu Li, Qinghui Luo, Zeyuan Xu, Xiaofei Li, Xuewei Qiao, Xinyi Xu, Xinni Song, Yao Wang, Yue’e Cao

**Affiliations:** 1 College of Resource and Environmental Science, Xinjiang University, Urumqi, Xinjiang, China; 2 Key Laboratory of Oasis Ecology of the Ministry of Education, Xinjiang University, Urumqi, Xinjiang, China; 3 Institute of Desert Meteorology, CMA, Urumqi, Urumqi, Xinjiang, China; Sun Yat-Sen University, CHINA

## Abstract

Knowledge about soil nitrogen (N) and phosphorus (P) concentrations, stocks, and stoichiometric ratios is crucial for understanding the biogeochemical cycles and ecosystem function in arid mountainous forests. However, the corresponding information is scarce, particularly in arid mountainous forests. To fill this gap, we investigated the depth and elevational patterns of the soil N and P concentrations and the N: P ratios in a *Picea schrenkiana* forest using data from soil profiles collected during 2012–2017. Our results showed that the soil N and P concentrations and the N: P ratios varied from 0.15 g kg^−1^ to 0.56 g kg^−1^ (average of 0.31 g kg^−1^), from 0.09 g kg^−1^ to 0.16 g kg^−1^ (average of 0.12 g kg^−1^), and from 2.42 g kg^−1^ to 4.36 g kg^−1^ (average of 3.42 g kg^−1^), respectively; additionally, values significantly and linearly decreased with soil depth. We did not observe a significant variation in the soil N and P concentrations and the N: P ratios with the elevational gradient. In contrast, our results revealed that the mean annual temperature and mean annual precipitation exhibited a more significant influence on the soil N and P concentrations and the N: P ratios than did elevation. This finding indicated that climatic variables might have a more direct impact on soil nutrient status than elevation. The observed relationship among the soil N and P concentrations and the N: P ratios demonstrated that the soil N was closely coupled with the soil P in the *P*. *schrenkiana* forest.

## Introduction

The theoretical relationship between foliar nutrient patterns and soil nutrients has been a cornerstone of ecosystem science for years [1–4]. The corresponding investigations have been conducted across different terrestrial ecosystems [5–8]. Among these studies, the majority have mainly focused on two categories of nutrients, i.e., macronutrients (e.g., nitrogen [N], phosphorus [P], and potassium [K]) and micronutrients (e.g., iron [Fe], manganese [Mn], and zinc [Zn]), based on their different functions in plant growth. Multiple studies have shown that soil macronutrients, particularly N and P, play vital roles in plant functioning and are among the most important limiting nutrients in terrestrial ecosystems [9–11]. Thus, their concentrations and mass ratios (N: P) are currently viewed as indices of the nutrient status that may provide insights into several processes, such as soil microbial activities, leaching, denitrification, and nutrient mineralization [12–14].

Worldwide, forest ecosystems represent the habitat of a considerable portion of our flora and fauna, and humans benefit from the diverse environmental functions of forests, such as climate, hydrology, water, air quality, and CO_2_ sequestration [15]. Soil N and P concentrations and N: P ratios of forests have been extensively investigated in recent years [16–18]. Currently, the issues, i.e., the influence of soil depth and elevation on forest soil N and P concentrations and N: P ratios and the response of the concentrations and the ratios to climatic variables, remain controversial, even though considerable research has been conducted [4, 19–21]. Therefore, a deep understanding of the patterns and drivers of forest soil N and P concentrations and N: P ratios is necessary to evaluate the sustainability of forest ecosystems, the cycling of N and P in forests, and the feedback of forest ecosystems in response to climate change [18].

Depth patterns of soil N and P concentrations and N: P ratios vary among different forest ecosystems. In a lower subtropical successional series in southern China and determined that the total N decreased with soil depth, the total P exhibited a complex variation among different forest species, and the N: P ratios of deep soils were higher than those of shallow soils [22]. Similar results were also reported in a study that focused on N and P stoichiometries across forest ecosystems in Northwest China [23]. In contrast, soil N and P increased with soil depth, and the N: P ratios increased from the upper to the lower soil horizons in subtropical plantations [24]. In a boreal forest of central Canada, the soil total N and P in surface mineral soil (0–15 cm) was lower than that in subsurface mineral soil (15–30 cm); however, the N: P ratios remained unchanged [25]. In a subalpine forest of central Nepal (Himalayan tree line), soil N and P concentrations and the N: P ratios consistently decreased with soil depth [21]. These results represent highly varied data for forest soil N and P concentrations and N: P ratios across different soil depths; this high variability highlights the importance of site-specific studies for the sufficient approximation of soil nutrients in forest ecosystems worldwide.

Plants could be limited by P in warm regions and by N in cold regions because of litter input, decomposition, leaching, and other factors [26–28]. This finding indicates the effect of temperature variation on the soil N and P concentrations and N: P ratios [27]. Experimental manipulations conducted in a grassland sufficiently demonstrated the response of the soil N and P concentrations and N: P ratios to variations in temperature [29]. For forest ecosystems, experimental manipulation is infeasible if the goal is to understand the response of N: P ratios to temperature variation (and other variables, e.g., precipitation). As a result, the soil N: P ratios along an elevational gradient have the potential to complement experimental manipulations. For example, the variation in climatic conditions could be determined through parameter measurements across sites [30]. To date, limited data are available on the variation in forest soil N: P ratios with changes in elevation. In alpine forest of the Eastern Tibetan Plateau, soil N: P ratios increased with elevation because of the high temperature and litter input at low elevations [31]. Similar results were reported in a study that aimed to understand the soil stoichiometric characteristics along elevational gradients in Southwest China [32]. Soil N: P decreased as elevation increased at Himalaya treeline [21]. Similar results were obtained in South American and African forest communities [33]. In *Picea crassifolia* forest of the Qilian Mountains near our study area, the soil N: P ratios initially increase and subsequently decrease with elevation, and the maximum ratio was found at 3100 m a.s.l. [34]. The authors interpreted this phenomenon as a result of the relatively low N concentrations and the high anthropogenic disturbance at low elevations. Overall, the patterns of the soil N: P ratios in forests along elevational gradients are far from being fully understood.

Apart from soil depth and elevation, most studies have focused on the relationship between soil N: P ratios and climatic conditions [18, 35–37]. In general, low temperature slows microbial activity and is responsible for the decrease in N decomposition from forest litter [18]. In contrast, high precipitation could result in strong leaching of the soil, which is consequently correlated with the low concentration of P in soils [18]. However, a wide variation among forest ecosystems has been observed. For example, a large-scale investigation of soil stoichiometry in forest ecosystems along the north-south transect in eastern China indicated that the soil N: P ratios increased with the mean annual temperature (MAT) and mean annual precipitation (MAP) [37]. In contrast, although the soil N and P showed a strong correlation with the MAT, the soil N: P ratios exhibited a weak relationship with the MAT across forests in China, additionally, soil N: P ratios increased along the precipitation gradient [38]. Another large-scale investigation observed a negative response of soil N: P ratios to MAT and a positive relationship between soil N: P ratios and MAP [18]. These studies have shed some light on the basic understanding of the spatial patterns of soil N: P. However, the exact response of the ratios to climatic variables remains unknown.

The Schrenk’s spruce (*Picea schrenkiana*) is one of the most widespread and important timber tree species in Central Asia. The distribution of Schrenk’s spruce covers an elevational range from 1,600 m to 2,700 m in the Tianshan Mountains, which is a large mountain system that lies 2,000 km from Uzbekistan, Kyrgyzstan, Kazakhstan, and China [39]. Being the largest latitudinal mountain system in the world, the Tianshan Mountains comprise the largest mountain system in the world that is found among arid regions and is far from the ocean [40]. Because the species spans such a wide elevational and longitudinal range, Schrenk’s spruce forest provides an opportunity to analyze the variation in soil N and P concentrations and N: P ratios across a wide elevational gradient and a gradient of MAT and MAP. The soil nutrient status of Schrenk’s spruce forests has been investigated [41–43]. However, no study has addressed the changes in soil N: P stoichiometric ratios in Schrenk’s spruce forest in relation to soil depth, MAT, MAP, and elevation across the distributional range of the species. In the present study, based on systematic field sampling from 2012 to 2017, we aimed to understand the response of soil N and P concentrations and N: P ratios to potential drivers (i.e., climate, soil, and elevation). Specifically, we aimed to (1) explore the effects of soil depth, MAT, MAP, and elevation on the patterns of soil N and P stoichiometries in Schrenk’s spruce forest and (2) identify the relationship between the N and P stocks and their stoichiometric ratios at different soil depths. By addressing these issues, this study provides a novel assessment of the spatial patterns of soil nutrients in a subalpine forest of an arid mountainous region.

## Materials and methods

### Study area

The study is not relevant to Human Subject Research, did not involved vertebrate animals, embryos or tissues, and endangered or protected species. No specific permissions were required for these sites we conducted soil sampling, some sites in tourist area could enter by tickets.

**Fig 1:**
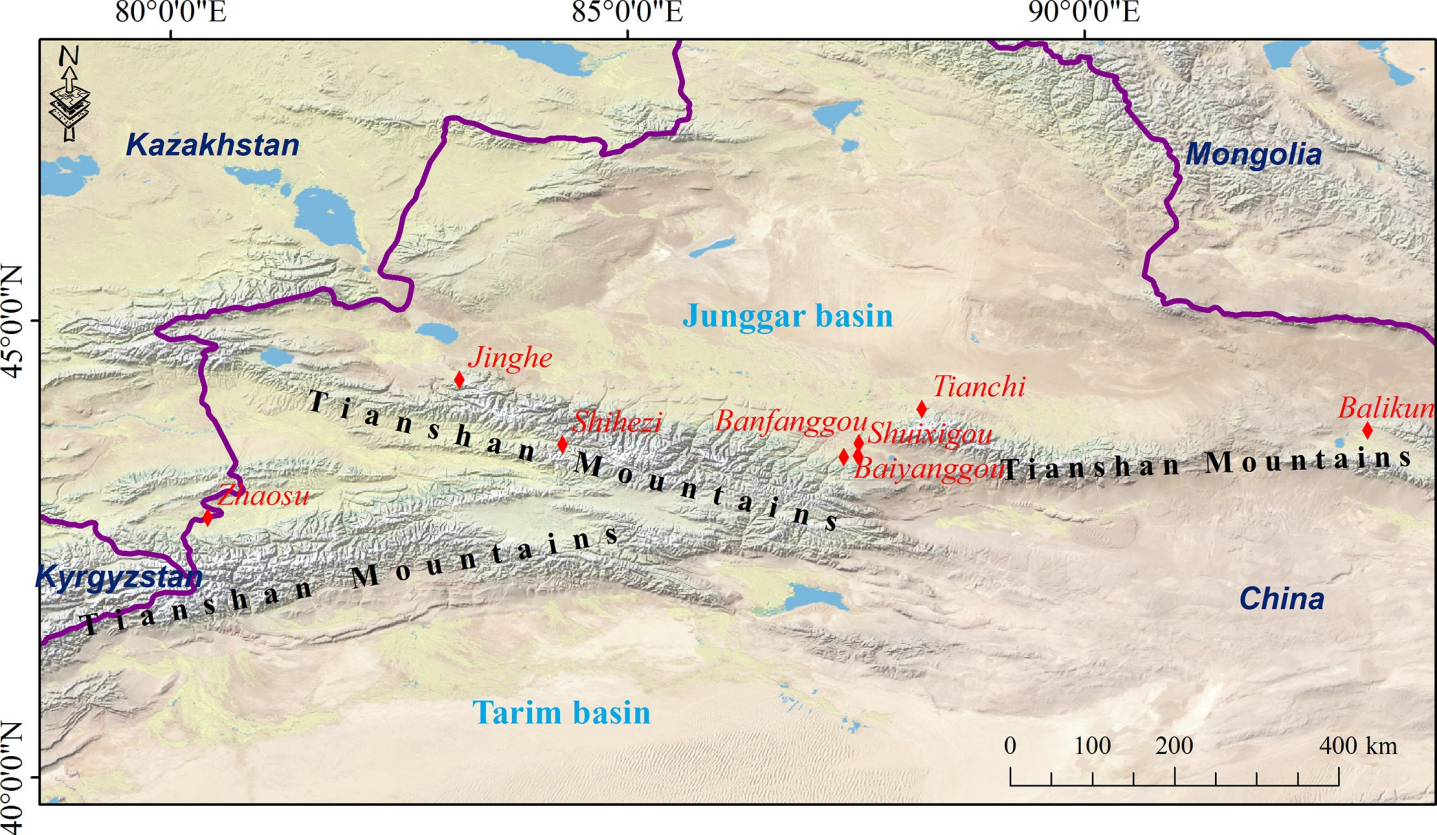
Location of the study area (Tianshan Mountains) and the sampling sites (red diamonds) in the Tianshan Mountains. The map with shaded relief was downloaded from Natural Earth (https://www.naturalearthdata.com/).

The present study was conducted in the Tianshan Mountains, which span from Uzbekistan to Northwest China, and the study comprised the western, central, and eastern parts. The Tarim and Junggar basins are located at the south and north of the Tianshan Mountains, respectively. The Ili River basin is situated between the western and central parts of the Tianshan Mountains (Fig 1). The study area is characterized by continental climate with cold and dry winters and relatively warm and humid summers, and more than 70% of precipitation occurs during the warm months, i.e., from May to September, because of the long distance from the ocean. In the Tianshan Mountains, the MAT decreases from 13.3°C at low elevations to -7.3°C at high elevations, and the MAP increases from less than 100 mm to more than 800 mm with the increase in elevation [44]. The vegetation types in Tianshan include (from low to high elevations) steppe, steppe-forest, subalpine shrubby meadow, alpine-frost action-barren zone, and permanent snow and ice [44]. Our field work was conducted in the steppe-forest belt on the northern slope of the Tianshan Mountains, where Schrenk’s spruce forest forms single-species stands between 1,600 m a.s.l. and 2,800 m a.s.l. Soils on the northern slope of the Tianshan Mountains mainly include leached yellowish brown soil and yellowish brown soil [45]. In spruce forest, the soil is deeper than 30 cm below the forest floor, and shallower than 30 cm at the upper and lower tree (Schrenk’s spruce) line [40].

### Field sampling and laboratory analysis

Sampling was conducted at eight sites (i.e., Zhaosu, Jinghe, Shihezi, Baiyanggou, Shuixigou, Banfanggou, Tianchi, and Balikun; Fig 1, No specific permissions were required for these sites where we conducted soil sampling) from June to September from 2012 to 2017. At each site, we selected slopes with Shrenk’s spruce forest and collected soil cores (we collected 90 cores in Schrenks’ spruce forest). Due to variation of soil depth, numbers of samples for each depth are different (90 for 0–10 cm and 10–20 cm depth, 85, 84, 83, 67, 59 and 39 for 20–30 cm, 30-40cm, 40–50 cm, 50–60 cm and 60–70 cm, respectively). During sampling work, we started at the plot (8-m radius circle) with the lowest elevation and ended at the plot with the highest location (up to the forest line of approximately 2,800 m), and elevational intervals were approximately 30 m. The longitude, latitude, and elevation of each plot were identified using a global positioning system receiver, i.e., eTrex venture. During sampling, soil samples from three random parallel profiles (within each plot) were collected from the top of the mineral soil to the top of the parent material in 10-cm intervals using a soil auger (1.5 cm). The parallel samples collected at each depth were mixed to obtain a composite sample. After being transported to the laboratory, the soil samples were air-dried and sieved. The soil total N concentrations (g kg^−1^) were analyzed using the Kjeldahl digestion method [46], and the total P concentrations (g kg^−1^) were measured using the perchloric acid digestion method followed by the molybdate colorimetric test [47].

### Climatic and topographic data

In this study, the climatic variables from the WorldClim 2 dataset (released in June 2016) were used [48]. This dataset contains the average monthly climate data for the minimum, mean, and maximum temperatures as well as precipitation data from 1970 to 2000. The dataset has different spatial resolutions (from 30 arc seconds to 10 arc minutes). We used the dataset with 30 arc seconds (approximately 1 km × 1 km), as this dataset has been evaluated and reliably used in forest science [49, 50]. Although selecting more variables guarantees a more comprehensive analysis, only the MAT and MAP were selected in the present study because of the existing collinearity among the variables [48]. Prior to the corresponding data analysis, these two variables were resampled (using bilinear interpolation) to 30 m × 30 m resolution for a detailed characterization of the MAT and MAP in the mountainous study area. The slopes of the sampling sites were calculated using the slope tool in the spatial analyst toolbox of ArcMap 10.0 (ESRI Inc). Specifically, we first calculated the slope (30 m × 30 m resolution) of the Tianshan Mountains and then extracted the slope value to each of the sampling sites. The cation exchange capacity (CEC, cmol_c_/kg), clay, sand and silt content (CC, SNC and SLC, respectively, mass fraction in %), soil pH and available soil water capacity (AWC, volumetric fraction) were extracted from the SoilGrids dataset. The SoilGrids provides global predictions for standard numeric soil properties (organic carbon, bulk density, Cation Exchange Capacity (CEC), pH, soil texture fractions and coarse fragments) at seven standard depths (0, 5, 15, 30, 60, 100 and 200 cm), the predictions are based on globally fitted models using soil profile and environmental covariate data.

### Statistical analysis

The total N and P stocks were calculated to discern their relationship with elevation. For each sampled soil profile, the N and P stocks were calculated as the sum of the product of soil bulk density (< 2 mm fraction in g cm^−3^), the N and P concentrations (g kg^−1^), and the soil layer thickness (cm) at each depth. All data were checked for homogeneity of variance and normality of distribution prior to analysis. ANOVA (one-way and two-way) was used to test the significant (p < 0.05) effect of the soil depth, elevation, MAT, MAP and combination of these factors (i.e., soil depth × elevation, soil depth × MAT, soil depth × MAP, elevation × MAT, elevation × MAP, and MAT × MAP) on the soil N and P concentrations and the N: P ratios. The correlation among the soil N and P concentrations and the N: P ratios with the soil depth, elevation, MAT, and MAP were analyzed using the Pearson correlation coefficient. Linear (simple and multiple) regression analysis was used to determine the linear relationship between the soil N and P concentrations and the N: P ratios with the soil depth, elevation, MAT, MAP, latitude, longitude and slope. All statistical analyses were performed using SPSS 19.0 (SPSS Inc., Chicago, IL, USA).

## Results

**Fig 2:**
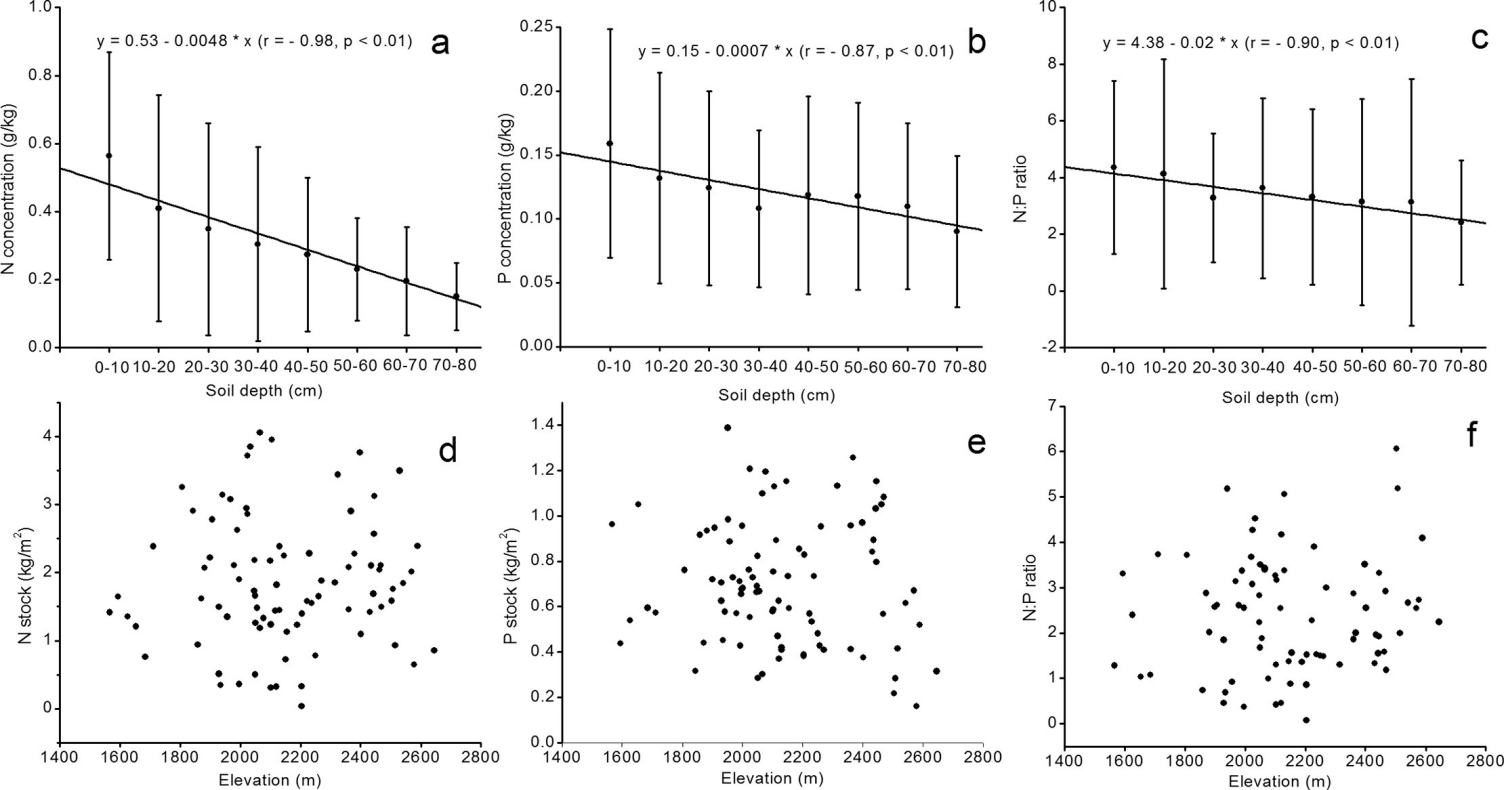
Relationship between N and P concentrations and N: P ratios with soil depth (a-c), and distribution of N and P stocks and N: P ratios with elevation (d-f).

At different soil depths, the N and P concentrations and the N: P ratios varied from 0.15 g kg^−1^ to 0.56 g kg^−1^ (average of 0.31 g kg^−1^), from 0.09 g kg^−1^ to 0.16 g kg^−1^ (average of 0.12 g kg^−1^), and from 2.42 g kg^−1^ to 4.36 g kg^−1^ (average of 3.42 g kg^−1^), respectively (Table 1). With the increase in soil depth, the N and P concentrations and the N: P ratios significantly and linearly decreased (Fig 2A–2C). No significant linear relationships were observed between the N and P stocks and the N: P ratios with elevation (Fig 2D–2F).

**Table 1:** N and P concentrations and N: P ratios at different soil depths.

	0–10 cm	10–20 cm	20–30 cm	30–40 cm	40–50 cm	50–60 cm	60–70 cm	70–80 cm
**N (g kg**^**−1**^**)**	0.56 ± 0.31	0.41 ± 0.33	0.35 ± 0.31	0.30 ± 0.28	0.27 ± 0.23	0.23 ± 0.15	0.20 ± 0.16	0.15 ± 0.10
**P (g kg**^**−1**^**)**	0.16 ± 0.09	0.13 ± 0.08	0.12 ± 0.08	0.11 ± 0.06	0.12 ± 0.07	0.12 ± 0.07	0.11 ± 0.06	0.09 ± 0.06
**N: P**	4.36 ± 3.05	4.13 ± 4.04	3.26 ± 2.29	3.63 ± 3.18	3.32 ± 3.09	3.15 ± 3.64	3.13 ± 4.35	2.42 ± 2.20

Value are expressed as the mean ± SD. The numbers of samples are 86, 87, 85, 84, 83, 67, 59, and 39 for the respective layers from 0–10 cm to 70–80 cm.

One-way ANOVA demonstrated that the influences of the soil depth, MAP, longitude and latitude on the soil N concentration were significant in Schrenk’s spruce forest (Table 2). For P concentration, the soil depth, longitude, latitude and slope were significant (Table 2). The soil depth, longitude and latitude were significant for the N: P ratios. Two-way ANOVA analysis demonstrated that interactions among factors without elevation (i.e., soil depth with MAT, soil depth with MAP and MAT with MAP) were significant for the N and P concentrations and the N: P ratios (Table 2).

**Table 2:** Results of ANOVA for effect of different variables on soil N and P concentrations and N: P ratios. One-way ANOVA: soil depth significant for N and P concentrations and N: P ratios; MAT significant for N: P ratios; MAP significant for N concentration; longitude and latitude significant for N and P concentrations and N: P ratios; slope of sampling plot significant for P concentration and N:P ratio. Two-way ANOVA: soil depth × MAT interaction significant for P concentration; soil depth × MAP interaction and MAT × MAP interaction significant for N and P concentrations and N: P ratios.

Factors	N concentration		P concentration		N: P ratio	
	*F-stat*	*p-value*	*F-stat*	*p-value*	*F-stat*	*p-value*
**Soil depth**	21.93	0.00**	4.99	1.16E-5**	2.08	0.04*
**Elevation**	0.76	0.74	0.67	0.79	2.73	0.12
**MAT**	1.01	0.48	0.89	0.62	1.07	0.05*
**MAP**	2.00	0.01**	1.22	0.25	1.45	0.13
**Longitude**	11.28	3.54E-8**	4.09	2.34E-3**	4.00	5.44E-3**
**Latitude**	4.37	3.07E-3**	3.89	6.14E-3**	3.73	2.83E-2*
**Slope**	0.38	0.76	7.88	1.03E-3**	2.06	0.13
**Soil depth × Elevation**	2.41	0.33	136.91	0.07	1.68	0.56
**Soil depth × MAT**	1.02	0.46	3.16	2.42E-4**	1.89	0.02*
**Soil depth × MAP**	2.18	6.99E-3**	5.57	2.48E-7**	1.76	0.03*
**Elevation× MAT**	0.03	1.00	4.18E-3	1.00	4.24	1.00
**Elevation × MAP**	0.04	1.00	4.78E-3	1.00	4.79	1.00
**MAT × MAP**	2.85	2.87E-3**	3.05	2.85E-3**	2.84	3.60E-3**

** statistical significance at p < 0.01

* statistical significance at p < 0.05.

**Fig 3:**
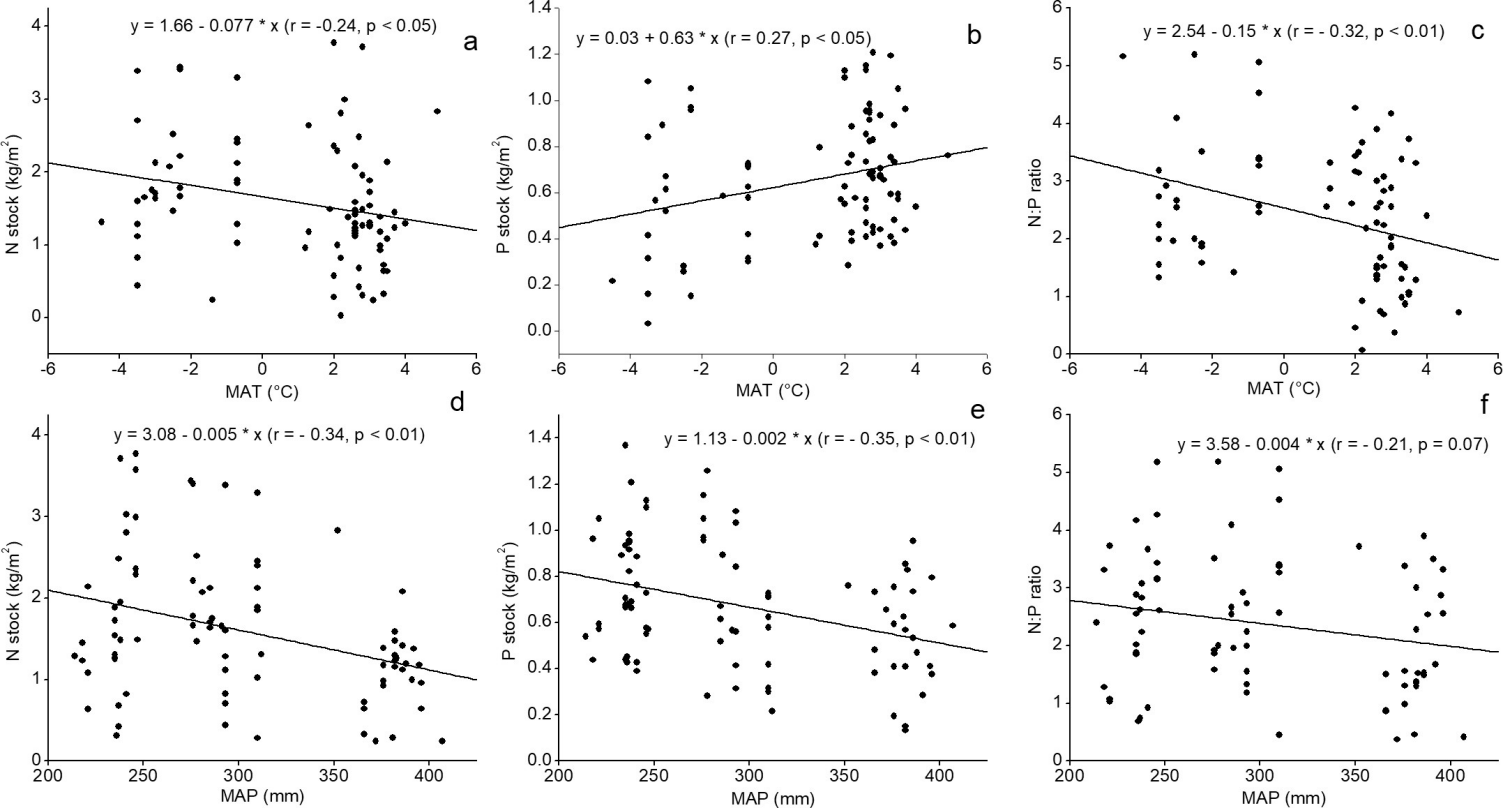
Relationship between the N and P stocks and N: P ratios with the MAT (a-c), and MAP (d-f).

**Fig 4:**
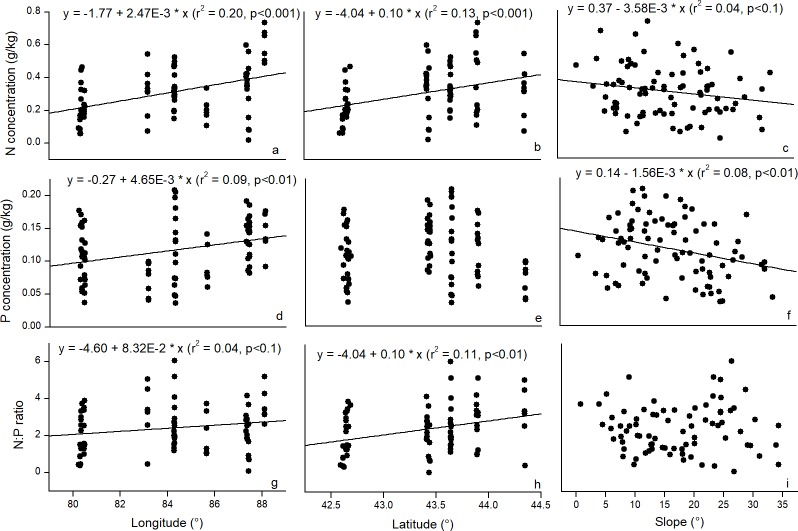
Relationship between the N and P concentrations and N: P ratios with longitude (a, d, g), latitude (b, e, h) and slope (c, f, i).

Simple linear regression indicated that the N stock and the N: P ratios significantly decreased with the MAT; however, the P stock increased with the temperature (Fig 3A–3C). The correlation coefficients between the N and P stocks and the N: P ratios with the MAT were -0.24, 0.27, and -0.32, respectively (Fig 3A–3C). Correspondingly, the MAT explained 5.7% (*r* = -0.24), 7.3% (*r* = 0.27), and 10.2% (*r* = -0.32) of the variation in the N and P stocks and the N: P ratios. The increase in the MAP at high elevations could result in the decrease in the N and P stocks and N: P ratios (Fig 3D and 3E). With a relatively high coefficient of determination, the influence of the MAP on the N (*r*^2^ = 0.12) and P (*r*^2^ = 0.12) stocks was stronger than that of the MAT. In contrast, the influence of the MAP on the N: P ratios (Fig 3F) was relatively weaker (*r* = -0.21) than that of the MAT (*r* = -0.32). Linear regression also indicated that the N and P concentrations and N: P ratios increased with longitude, which explained 20%, 9% and 4% of the variations in the N concentration, P concentration and N: P ratios (Fig 4A, 4D and 4G), respectively. Latitude explained 13% and 11% of the variation in the N concentration and the N: P ratios (Fig 4B and 4H). As the slope increased, the N and P concentrations decreased linearly (Fig 4C and 4F).

Multiple linear regression indicated that the linear combinations of elevation and MAP, MAT and MAP, and elevation and MAT and MAP explained 21%, 21% and 21% of the variation in the N concentration, respectively; however, the combination of elevation and MAT only explained 4% of the variation in the N concentration (Table 3). For the P concentration, although the regression relationship was significant at the 0.05 level, the combination of elevation and MAT only explained 10% of the variation in P. Similarly, if included elevation as an independent variable to determine the response of the N: P ratios to impact factors, a relatively lower coefficient of determination and F-value were obtained (*r*^2^ of 0.04 and F-value of 1.83 for the combination of elevation and MAP, as shown in Table 3).

**Table 3:** Results of multiple regression analysis between dependent variables (N and P concentrations and N: P ratios) and independent variables (elevation, MAT and MAP).

Independent variable	Regression coefficients of dependent variables			Interception	N	*r*^2^	*F-stat*
	Elevation	MAT	MAP				
**N concentration**	-1.41E-4	-0.02		0.63	81	0.04	1.65
	9.84E-5		-1.28E-3	0.48		0.21	10.54**
		-7.67E-3	-1.14E-3	0.66		0.21	10.21**
	8.13E-5	-2.01E-3	-1.26E-3	0.51		0.21	6.95**
**P concentration**	-6.31E-6	-7.77E-3		0.15	76	0.10	4.06*
	8.76E-5		-3.98E-4	0.06		0.21	9.49**
		-7.15E-3	-2.90E-4	0.23		0.20	9.18**
	5.94E-5	-3.94E-3	-3.56E-4	0.13		0.22	6.81**
**N: P ratio**	-2.18E-3	-0.31		7.93	85	0.12	5.44**
	4.70E-4		-6.42E-3	3.86		0.04	1.83
		-0.17	-5.49E-3	4.75		0.10	4.43*
	-1.74E-3	-0.28	-2.63E-3	7.73		0.12	3.79*

** statistical significance at p < 0.01

* statistical significance at p < 0.05.

As shown in Table 4, the N concentration showed a positive correlation with the soil CEC and SC, with correlation coefficients of 0.25 (p < 0.05) and -0.29 (p < 0.01), respectively. Although insignificant (p = 0.10), the P concentration was positively correlated with the CEC (Table 4). The N: P ratios were positively correlated with the CC (p < 0.05).

**Table 4:** Correlations (Pearson’s *r*) among soil N and P concentrations and N: P ratio with parameters of parent materials.

	CEC	CC	SNC	SLC	pH	AWC
**N concentration**	0.25*	0.20	0.10	-0.29**	-0.04	-0.03
**P concentration**	0.18	3.61E-3	0.04	-0.05	-0.13	-0.06
**N: P ratio**	0.00	0.23*	0.02	-0.19	0.06	-0.1

CEC, cation exchange capacity (cmol_c_/kg); CC, clay content (%); SNC, sand content (%); SLC, silt content; AWC, available soil water capacity (volumetric fraction in cm^3^·cm^−3^).

Significance levels are given as **

p < 0.01; *

p < 0.05; no symbol, p > 0.05.

The correlations between the N and P stocks at the different soil depths were positive and significant (Table 5). At the surface (i.e., 0–10 cm) and parent material (i.e., 60–70 and 70–80 cm) soils, the correlations between the N and N: P ratios were weak. The determination of the P concentrations on the N: P ratios was higher than that of N (only at the 20–30 cm layer, the correlation between the N and N: P ratios was higher than that of the P and N: P ratios, as shown in Table 5). Pearson’s *r* between N and P increased with soil depth, indicating a closer relationship between N and P in deep soils than in shallow soils (Table 5). In contrast, Pearson’s *r* between the N and N: P ratios and between the P and N: P ratios exhibited no differences among the different soil depths (Table 5).

**Table 5:** Correlations (Pearson’s *r*) among soil N and P concentrations and N: P ratios at different soil depths.

Soil depth	0–10 cm		10–20 cm		20–30 cm		30–40 cm		40–50 cm		50–60 cm		60–70 cm		70–80 cm	
Component	P	N: P ratios	P	N: P ratios	P	N: P ratios	P	N: P ratios	P	N: P ratios	P	N: P ratios	P	N: P ratios	P	N: P ratios
**N**	0.22*	0.11	0.24**	0.06*	0.38**	0.49**	0.46**	0.27*	0.31**	0.35**	0.21	0.27*	0.33*	0.04	0.51**	0.07
**P**		-0.47*		-0.42**		-0.37**		-0.45**		-0.47**		-0.53**		-0.34**		-0.45**

Significance levels are given as **

p < 0.01

*, p < 0.05

no symbol, p > 0.05.

## Discussion

### N and P concentrations and N: P ratios with soil depth

**Fig 5:**
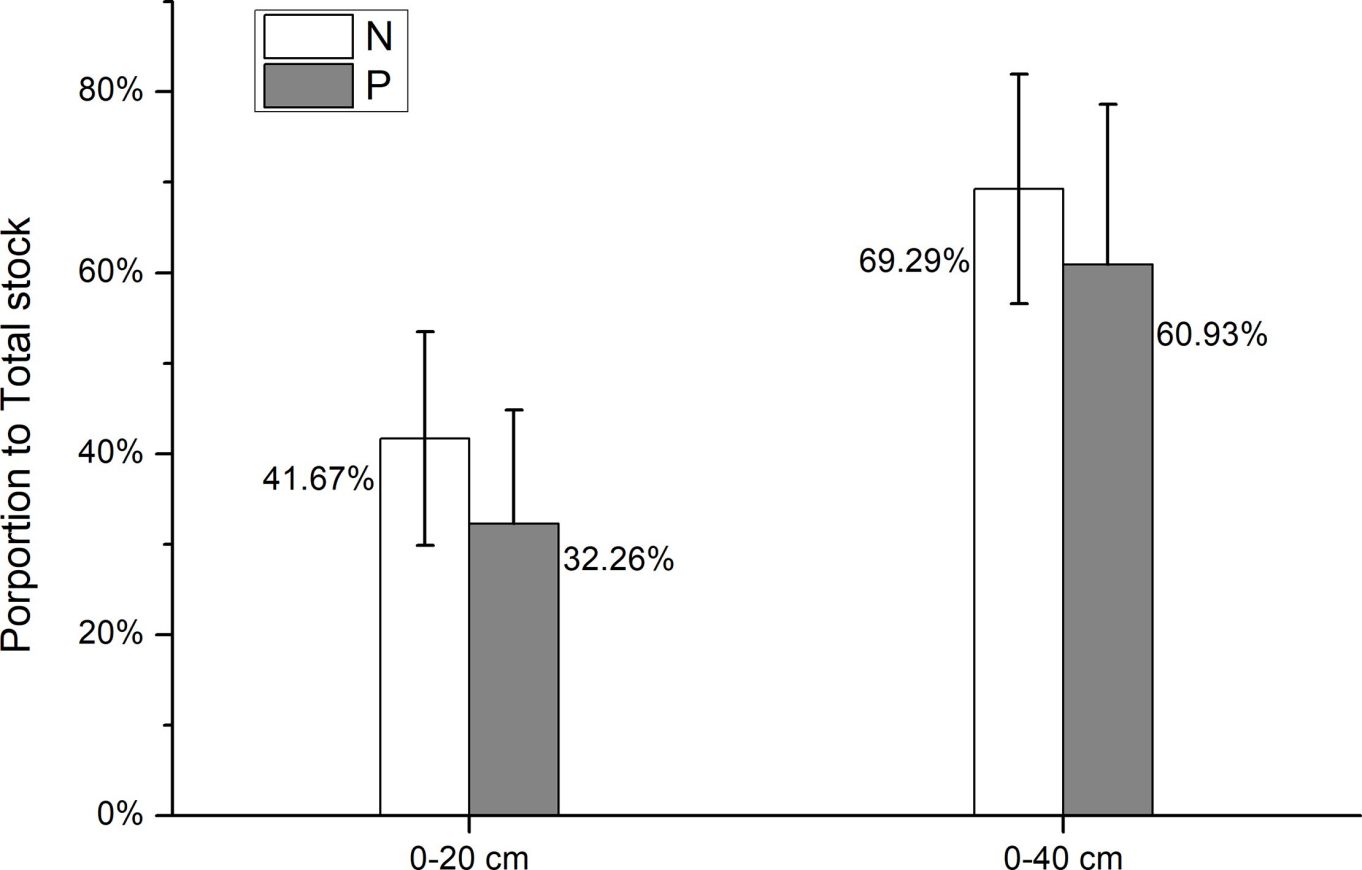
Average proportion of N and P stocks in the 0–20 and 0–40 cm soils to the stocks in the entire soil profile.

To our knowledge, this study is the first to investigate the depth pattern of soil N and P concentrations and N: P ratios in Schrenk’s spruce forest. Our results showed that the soil N stock ranged from 0.03 kg m^−2^ to 4.05 kg m^−2^, with an average of 1.84 ± 0.94 kg m^−2^. The soil N significantly decreased with soil depth (Fig 2 and Table 1), indicating a high concentration of N in shallow soils. Specifically, we calculated the proportion of the N stock in the upper soils (0–20 and 0–40 cm) to that in the entire soil profile. The results showed that 41.67% and 69.29% of N were stored in the surface 0–20 and 0–40 cm soils, respectively (Fig 5). In general, N decreased with the increase in soil depth in forest ecosystems [22, 33, 51, 52]. A few exceptions found no relationship between the N concentration and the soil depth due to the disturbance caused by human activities in planted forests [53–55]. The P stock in Schrenk’s spruce forest ranged from 0.16 kg m^−2^ to 1.38 kg m^−2^, with an average of 0.70 kg m^−2^. It should be noted that in the present study, the P concentration was the total P concentration that was obtained by the perchloric digestion method. Since there are two essential P cycles in forest soils (i.e., the inorganic cycle driven by leaching and acid formation, the fast cycling, and the biological cycle driven by plants and microbes, the slow cycling), the P content in soils may vary due to variations in the inorganic and biological cycles.

Similar to the N stock, the P concentrations and stock in Schrenk’s spruce forest also significantly decreased with soil depth (Fig 2 and Table 1). This result is in accordance with that of a pioneering synthesis work, which discussed the variation in element stocks of forest soils from local to global scales [56]. Recent investigations conducted in different forest ecosystems have also confirmed such reductions in P concentrations with soil depth [55, 57, 58]. Given that plant uptake may play a role in determining the soil nutrient status at different spatial and temporal scales [59], particularly in arid environments [60], the decrease in the soil N and P concentrations with soil depth in Schrenk’s spruce forest could be primarily explained by the root characteristics of this species. Since Schrenk’s spruce is a shallow-root species (the roots of Schrenk’s spruce are found at depths shallower than 1 m) with more fine roots in deep soils [61], the corresponding increase in the nutrient uptake resulted in the decrease in the N and P concentrations. By comparing the N and P concentrations at different soil depths, we found that the variation in the N concentrations was higher than that of the P concentrations, especially in deep soils. Similarly, variation in N was higher than that in P in shallow soils in a subtropical forest [62], suggested that the supply of P from weathering of the parent material may also affect the depth distribution of soil P in forest ecosystems [18, 63, 64].

The N: P ratios decreased with soil depth, as shown in Fig 2 and Table 1. Given that the N and P concentrations decreased, the decrease in the N: P ratios with soil depth indicated the dominance of N on the ratios. Actually, N decreased from 0.56 g kg^−1^ in 0–10 cm soil to 0.15 g kg^−1^ in 70–80 cm soil, which indicated a 73.21% decrease in the concentrations; furthermore, this decrease was higher than that of P, which had a 43.75% decrease (0.16 g kg^−1^ to 0.09 g kg^−1^ from 0–10 cm to 70–80 cm soils). Similar results were also reported in [65], where the soil P concentration decreased more slowly than did the N concentration. This could be because (1) there was an accumulation of nutrients at the surface caused by the return of dead biomass debris, (2) the leaching was the result of water infiltration, and (3) the P supply from weathered parent materials was higher than the supply of N [6].

### N and P concentrations and N: P ratios with elevation

Our data showed no significant response of the soil N and P stocks to elevation (Fig 2D and 2E). Contrasting influences of elevation on N and P stocks have been reported in different forest ecosystems. For instance, soil N stock was highly positively correlated with the elevation in subtropical open forests [66]. Similarly, significantly more N was stored in the high-elevation northern hardwood soil than in the low-elevation soil [67]. However, in a northern hardwood forest ecosystem, the soil N stock was not correlated with the elevation [68]. The soil P stock in forest ecosystems might be positively [69, 70] or negatively [71–74] correlated with the elevation. In contrast, no significant differences in the P stocks among soils obtained along an elevational gradient in tropical rainforest [75].

The previously outlined correlation of the soil N and P stocks with elevation gives us a sense of the varied responses of the N: P ratios to elevation in forest ecosystems. Actually, in an assessment of the soil N and P stoichiometric characteristics and their impact factors along an elevational gradient in Southwest China, soil N: P ratios were positively correlated with the elevation [32]. Similar results were obtained in a subalpine forest on the southern slope of the Himalayan Mountains [21]. In contrast, the ratios decreased significantly with the increase in the elevation in forests of the karst area [76]. In semiarid Qilian Mountains of China, N: P ratios of soil initially increased and subsequently decreased with the increase in elevation [34]. Similar trends were observed in tropical and subtropical monsoon forests of China [77]. The initial increase in the N: P ratios might be due to the increase in N provided by the litter input and microbial decomposition, which corresponds to the increase in precipitation with the increase in elevation to a certain elevation, i.e., 3100 [34]. In contrast, the subsequent decrease in the N: P ratios may indicate the increase in the P supply from parent materials that was stimulated by the increase in precipitation and the decrease in temperature.

The non-significant relationships between the N and P stocks and the N: P ratios with elevation in *P*. *schrenkiana* forest, as well as in other forests, indicated that the elevation may not be the main factor that influences the variation in nutrient stocks and stoichiometry in forest ecosystems, especially forests in arid mountainous regions. In general, the decrease in soil depth with the increase in elevation could reduce the stock of nutrients [34, 78]. In addition, the average N and P concentrations at high elevations were generally lower than those at low elevations [21, 68, 79]. However, in our study area, we did not observe significant differences in the N and P concentrations and soil depth along the elevational gradient (S1 and S2 Figs). Moreover, because changes in many other factors could influence the soil N and P stocks with elevation, the relatively stable value of the N and P stocks in Schrenk’s spruce across the elevational range was unexpected.

### N and P concentrations and N: P ratios with MAT and MAP

A thorough understanding of the influence of climatic variables on nutrient stocks and biological stoichiometry provides a basis for determining the causes of nutrient cycling in terrestrial ecosystems [80]. Our results showed that, with the increase in MAT, the N stock decreased (Fig 3A). This result contrasts with the generally accepted relationship between the MAT and the soil N stock; thus, these results demonstrated that, with the increase in the mass production and litter accumulation and with the strengthened decomposition activity of microbes under warm conditions, the N stock increased with the increase in the MAT [81, 82]. In our study area, the decrease in the N stock with the increase in the MAT indicated the high N uptake of trees under warm conditions [74]. In contrast, more N was deposited due the combined effects of the reduced decomposition rate and the suppressed plant uptake. Compared with the decrease in soil N with the increase in MAT, the soil P was higher under warmer conditions, which suggested that the supply of P from the parent material compensated for the loss of the element through plant uptake. Theoretically, parent material weathering was enhanced under warmer conditions [83].

The soil N and P decreased as MAP increased, indicating precipitation has a negative effect on the N and P stocks in Schrenk’s spruce forest. In Schrenk’s spruce forest, a high MAP actually corresponded to a high elevation. No significant differences were observed in the N and P concentrations and soil depth with the elevation (S1 and S2 Figs); thus, the uptake by trees might be the main factor that influenced the decrease in the N and P stocks. Specifically, high precipitation accelerated the growth rate of trees [84, 85], consequently resulting in low soil N and P stocks. This type of relationship between precipitation and soil nutrient status has been confirmed in other forest ecosystems in arid regions, such as the Loess Plateau [86, 87], the Qinling Mountains [88] of Northwest China, and the Qilian Mountains near our study area [89].

Our results indicated that the N: P ratios decreased as MAT increased (Fig 3C), which was inconsistent with the large-scale studies [38]. Similar with our study, low N: P ratios at high temperatures were reported [64, 90]. In addition, the soil N: P ratios in Schrenk’s spruce forest also exhibited a decreasing trend as MAP increased (Fig 3F). In general, soil P decreased faster than did soil N with the increase in precipitation, resulting in higher N: P ratios along the precipitation gradient. Large-scale studies have also confirmed such patterns in N: P ratios, i.e., relatively lower availability of P than N due to the high rate of P leaching in tropical and subtropical forests and relatively higher availability of P than N due to the low rate of P leaching in arid environments [91]. In the present study, the decrease in the N: P ratios with the MAT and MAP probably indicated that N was the limiting element for biomass accumulation in Schrenk’s spruce. This finding was consistent with the results of other large-scale investigations [9, 27]. Actually, the soil N: P ratios in Schrenk’s spruce forest (2.39) were not only lower than the average of the global forest soil [92] but also lower than that of a mountainous forest near our study area [34]. As a result, the warm (high MAT) and wet (high MAP) conditions stimulated biomass accumulation in the species, thereby resulting in the high N uptake of trees from the soil and finally contributing to the low N: P ratios of the soils.

### Correlation among soil N and P concentrations and N: P ratios

The soil N concentrations were positively correlated with the N: P ratios in the Schrenk’s spruce forest (Table 4), which was consistent with the results of other studies [4, 93, 94]. We also found that the relationship (N concentrations and N: P ratios) diminished in deep soils (60–70 and 70–80 cm, as shown in Table 4). The diminished relationship may indicate that the N: P ratios at these two layers have no significant response to the N concentrations. The possible cause may include the following. First, it could be due to the relatively stable (lower variation) N concentrations at these two layers (Fig 2A). Second, it could be due to the varied P concentrations that resulted from the P supplied by the weathering of the parent material.

The soil N and P concentrations were positively correlated. The correlation coefficient ranged from 0.21 to 0.51. Most of the correlations were significant at the 0.05 level. This significant positive relationship demonstrated that the soil N was closely coupled with the soil P in the Schrenk’s spruce forest. The observed relationship between the N and P concentrations was consistent with previous findings derived from foliar [9, 34, 95] and microbial communities [80, 96, 97], which jointly indicated the coherent characteristics of elemental cycles in different components within terrestrial ecosystems. Although a negative relationship between the soil N and P in *Betula platyphylla*-planted forest was reported, this was probably due to the influence of human activities [54].

## Concluding remarks

In this study, we examined the soil N and P concentrations and the N: P stoichiometric ratios in Schrenk’s spruce forest of Northwest China using data from soil profiles collected from eight sites during field work conducted from 2012 to 2017. In line with other forest ecosystems, our results indicated that the soil N and P concentrations and the N: P ratios in Schrenk’s spruce forest decreased with the soil depth. In contrast to other studies, we did not observe a clear trend in the N and P concentrations and N: P ratios with the increase in elevation. Notably, our results showed that the N and P stocks and N: P ratios were more significantly correlated with MAT and MAP than with elevation. This finding indicated that MAT and MAP have direct influences on the elemental stock and biogeochemical cycles in arid mountainous forests. In addition, linear regression indicated that the N and P concentrations and N: P ratios increased with longitude and latitude, and the N and P concentrations decreased linearly with slope. Furthermore, the positive correlation between the soil N and P in Schrenk’s spruce forest was consistent with that reported elsewhere, which jointly demonstrated that the soil N was closely coupled with the soil P in forest ecosystems.

## Supporting information

S1 Fig Distribution of N and P concentrations with elevation. (TIF)

S2 Fig Distribution of soil depth along the elevational gradient. (TIF)

S1 File The data collected and analyzed in this study. (RAR)
