# Correction: Climatic and topographic variables control soil nitrogen, phosphorus, and nitrogen: Phosphorus ratios in a *Picea schrenkiana* forest of the Tianshan Mountains

**DOI:** 10.1371/journal.pone.0211839

**Published:** 2019-01-30

**Authors:** Zhonglin Xu, Yapeng Chang, Lu Li, Qinghui Luo, Zeyuan Xu, Xiaofei Li, Xuewei Qiao, Xinyi Xu, Xinni Song, Yao Wang, Yue’e Cao

There is an error in the fourth sentence of the Abstract. The correct sentence is: Our results showed that the soil N and P concentrations and the N: P ratios varied from 0.15 g kg^−1^ to 0.56 g kg^−1^ (average of 0.31 g kg^−1^), from 0.09 g kg^−1^ to 0.16 g kg^−1^ (average of 0.12 g kg^−1^), and from 2.42 to 4.36 (average of 3.42), respectively; additionally, values significantly and linearly decreased with soil depth.
